# Time-Restricted Feeding and Metabolic Outcomes in a Cohort of Italian Adults

**DOI:** 10.3390/nu13051651

**Published:** 2021-05-13

**Authors:** Walter Currenti, Silvio Buscemi, Raffaele Ivan Cincione, Achille Cernigliaro, Justyna Godos, Giuseppe Grosso, Fabio Galvano

**Affiliations:** 1 Department of Biomedical and Biotechnological Sciences, University of Catania, 95123 Catania, Italy; currentiw@gmail.com (W.C.); justyna.godos@gmail.com (J.G.); fgalvano@unict.it (F.G.); 2 Biomedical Department of Internal and Specialist Medicine (DIBIMIS), University of Palermo, 90123 Palermo, Italy; silvio.buscemi@unipa.it; 3 Department of Clinical and Experimental Medicine, University of Foggia, 71122 Foggia, Italy; ivan.cincione@unifg.it; 4 Department of Health Service and Epidemiological Observatory, Health Authority Sicily Region, 90145 Palermo, Italy; achille.cernigliaro@regione.sicilia.it

**Keywords:** time-restricted feeding, fasting, intermittent fasting, dyslipidemia, metabolic, obesity, hypertension, cohort, Mediterranean diet, breakfast

## Abstract

Background: research exploring the effects of food timing and frequency on health and disease is currently ongoing. While there is an increasing body of scientific literature showing the potential health benefits of intermittent fasting (IF) in laboratory settings and in animals, studies regarding IF on humans are limited. Therefore, the objective of this research was to evaluate the relationship between the feeding/fasting time window and metabolic outcomes among adult individuals. Methods: dietary and demographic data of 1936 adult subjects living in the south of Italy were examined. Food frequency questionnaires (FFQ) were administered to determine the period of time between the first and the last meal of a typical day. Subjects were then divided into those with a time feeding window lasting more than 10 h, within 8 h (TRF-8) and within 10 h. Results: after adjustment for potential confounding factors related to eating habits (such as adherence to the Mediterranean diet, having breakfast/dinner), TRF-10 was inversely associated with being overweight/obese (OR = 0.05, 95% CI: 0.01, 0.07), hypertension (OR = 0.24, 95% CI: 0.13, 0.45), and dyslipidemias (OR = 0.26, 95% CI: 0.10, 0.63), while TRF-8 only with being overweight/obese (OR = 0.08, 95% CI: 0.04, 0.15) and hypertension (OR = 0.33, 95% CI: 0.17, 0.60). No associations were found with type-2 diabetes. Conclusions: individuals with a restricted feeding time window were less likely to be overweight, obese and hypertensive. Further studies are needed to clearly validate the results of the present study.

## 1. Introduction

In the last decades, obesity and overweightness have become among the major public health issues considering their constant increasing prevalence in both developed and developing countries [1,2]. This dramatic increase in obesity is a result of radical social and economic changes since the early 20th century in Western countries, which has led to the establishment of an “obesogenic environment” characterized by urbanization, industrialization, economic growth, mechanized transportation and the growing availability of abundant, inexpensive, and often nutrient-poor food [3]. Obesity is generally caused by an energy imbalance between consumed calories and expended calories; however, not all people living in an obesogenic environment express an excessive weight gain; thus, other factors such as genes, hereditary elements, race/ethnicity, microbiome, family history, drugs, comorbidity, viral infections and, especially, general lifestyle can influence the body’s response to food intake [4]. The public health interest toward obesity relies on the fact that it represents a primary risk factor for many disorders, especially type 2 diabetes, cancer, and cognitive and cardiovascular diseases [5].

Recent evidence has highlighted the role of circadian rhythm in controlling human dietary patterns, behavior and metabolism [6]. The circadian system is characterized by cyclical endogenous processes that last approximately 24 h and are regulated by the suprachiasmatic nucleus (SCN), located in the anterior hypothalamus of mammals. The SCN clock is mainly influenced by the cyclicality of light and dark; light triggers photosensitive retinal ganglion cells, especially the photopigment melanopsin, and reaches the suprachiasmatic nucleus via the retinohypothalamic tract [7]. The presence of circadian clocks has been demonstrated not only in the SNC, but also in peripheral tissues and in other brain areas.

In fact, the human expression of the circadian clock genes has been found in many organs, such as the heart, liver, muscle, kidneys, pancreas, adipose tissue and lungs. Food appears to be the most relevant factor capable of synchronizing the peripheral circadian clocks, and postponed feeding due to prolonged nighttime wakefulness leads to a desynchronization between central and peripheral circadian clocks [8].

Dissimilarly from the unintentional time-restricted feeding habits adopted by our ancestors for millennia, nowadays, the prevailing nutritional habit is to ingest most of the caloric ingestion later in the evening hours [9]. Over the last few years, many studies have focused on the timing of food intake in the context of circadian rhythms: time-restricted feeding (TRF) refers to the restriction of dietary intake within a relatively short window of time (generally less than 10 h) without altering nutrient quality or calories [10]. There is emerging evidence that TRF might have a positive impact on metabolic health [11], including influencing body composition, lipid profile, insulin levels, blood pressure and oxidative stress [12]. Moreover, restriction of feeding time early in the day (i.e., having early supper and skipping dinner) has been reported to further positively affect metabolic outcomes compared to late TRF (i.e., skipping breakfast or evening eaters for religious matters) because it is in better alignment with the circadian rhythm [13]. Although the hypothesis of applying timing rather than caloric restriction is attractive, current evidence is too scarce to draft conclusions. Thus, the objective of this study was to examine the relationship between the time feeding window duration and various metabolic outcomes in a population-based cohort of adult subjects living in the Mediterranean area of Italy.

## 2. Materials and Methods

### 2.1. Study Population

This is a cross-sectional study conducted on the participants of the Mediterranean healthy Eating, Aging, and Lifestyles (MEAL) study, an observational study aiming to evaluate the association between dietary and lifestyle factors and health outcomes in subjects living in the south of Italy. Between 2014 and 2015, a random sample of 2044 adult (age 18+ years) men and women was enrolled in the urban area of Catania, the second biggest city of Sicily, southern Italy. Full details about the study protocol (rationale, design and methods) have been reported elsewhere [14]. Inclusion criteria were the following: (i) age > 18 years old, (ii) born and resident in Sicily, and (iii) Caucasian race; exclusion criteria were (i) serious disabling diseases (i.e., terminal illness, chronically bedridden patients, etc.), (ii) mental illness, and (iii) pregnancy. The study procedures were carried out in accordance with the Declaration of Helsinki (1989) of the World Medical Association. Written informed consent was obtained from the participants and the study protocol was agreed by the ethics committee of the referent health authority.

### 2.2. Data Collection

Demographic (i.e., age, sex, occupational and educational level) and lifestyle information (i.e., smoking habits, physical activity) was investigated. Occupational level was divided into four categories: (i) unemployed, (ii) unskilled workers, (iii) partially skilled workers, and (iv) skilled workers. Educational level was classified into three categories: (i) primary/secondary school, (ii) high school, and (iii) university. The physical activity level was assessed through the International Physical Activity Questionnaires (IPAQ) [15], which consist of questions regarding five areas on time spent engaged in physical activity in the last week: “high physical activity level” is considered as performing strenuous physical activity on no less than three days, reaching a minimum total physical activity of no less than 1500 MET minutes per week or ≥ seven days of any combination of walking, moderate intensity or vigorous intensity activities reaching a minimum total physical activity of no less than 3000 MET minutes per week, while “moderate physical activity level” is considered as performing ≥ three days of vigorous intensity activity and/or walking of no less than 30 min a day or ≥ five days of moderate intensity activity and/or walking of no less than 30 min a day or ≥ five days of any combination of walking, moderate intensity or vigorous intensity activities reaching a minimum total physical activity of no less than 600 MET minutes per week. Finally, participants who did not meet the criteria before for either moderate or high levels of physical activity were categorized as having a low physical activity level [15]. Smoking status was divided into three categories: (i) non-smoker, (ii) ex-smoker, and (iii) current smoker.

### 2.3. Metabolic Outcomes

Anthropometric parameters were carried out according to a standardized protocol [16]. Height was assessed barefoot, with the back square against the wall tape, eyes looking straight ahead, and a right-angled triangle resting on the head and against the wall approximating to the nearest 0.5 cm. Subjects were classified according to body mass index (BMI) categories as under/normal weight (BMI < 25 kg/m^2^), overweight (from BMI 25 to 29.9 kg/m^2^), and obese (BMI ≥ 30 kg/m^2^). After 5 min sitting at rest, individual’s arterial blood pressure was checked three times on the right arm with an angle of 45 degrees from the trunk. The mean value of the last two measurements was considered as the final result. Data from measurements were expanded with general practitioners’ digital records, as a specialist diagnoses patients with disease in order to obtain drugs. Subjects were diagnosed with hypertension when the average systolic/diastolic blood pressure value was equal or more than 140/90 mm Hg (in accordance with the European Society of Cardiology (ESC)/European Society of Hypertension (ESH) guidelines) [17], taking anti-hypertensive medications, or being diagnosed previously as hypertensive. Individuals were considered dyslipidemic or diabetic if diagnosed with hypercholesterolemia/hypertriglyceridemia or diabetes, respectively.

### 2.4. Dietary Assessment

Nutritional data were obtained through two food frequency questionnaires (long and short), conceived and before validated for the Sicilian population [18,19]. The FFQs is composed by 110 drink and food items indicative of the diet in the last six months. Subjects enrolled were asked how often they usually had consumed pre-defined serving sizes of foods and beverages present in the FFQs, with a range of nine possible answers from “never” to “4–5 times per day”. Intake of food items characterized by seasonality referred to consumption during the period in which the food was available and then adjusted by its proportional intake over one year. The food consumption was assessed using a constructed database and calculated (in g or mL) by following the standard portion sizes used in the study and then turned into 24-h intake; then, food composition tables from the Italian Research Center for Foods and Nutrition to retrieve the mean content values for macro- and micronutrients contained in the FFQs were used. The nutrient intake from each food was obtained by multiplying the retrieved content by the consumption of each food daily. After the dietary assessment, individuals with unreliable intakes (<1000 or >6000 kcal/day, lacking food items or implausible responses) were excluded, leaving a total of 1936 subjects included in the analyses for the present study. The Mediterranean diet adherence was estimated through a literature-based score built weighting all the median (or mean) values for the sample size of selected studies published on Mediterranean diet and health outcomes and then determining a mean value of all the weighted medians; two standard deviations were used to determine three different categories of consumption for each food group for which a score depending whether they are representative of the Mediterranean dietary habits (fruit, vegetables, cereals, legumes, seafood, moderate intake of alcohol) or not (meat, preserved meats and dairy products) was given [20,21].

Adherence to the Mediterranean diet was evaluated through a validated, literature-based score not considering the consumption of nuts among its criteria [20]. The score was built reviewing available literature investigating the relationship between Mediterranean diet adherence and health outcomes, and based on the consumption of nine food groups, including fruit, vegetables, cereal grains, legumes, fish and fish products, meat and meat products, dairy products, alcohol intake and olive oil. Higher consumption of peculiar food groups of the Mediterranean diet (fruit, vegetables, cereals, legumes and fish) was given higher points, while higher consumption of food groups not representing it (meat and dairy products) was given lower points. The final adherence score varies from 0 point (lowest adherence) to 18 points (highest adherence) with individuals grouped in tertiles and categorized as low, medium, and high adherent to the Mediterranean diet [21].

### 2.5. Time Feeding Assessment

Participants were asked how many meals they had per day and at what time they usually had them during the last six months (including data about breakfast, snacks, lunch and dinner). Consequently, the time between the first and last meal of an average day was determined. Two categorizations for two groups of analyses were used: participants were categorized (1) in those eating within 10 h (TRF-10) vs. those eating over a window of time larger than 10 h (no TRF-10), and (2) in those eating within 8 h (TRF-8) vs. those eating over a window of time larger than 8 h (no TRF-8). These two windows of time were considered in order to test whether there could be some differences when exploring a narrower feeding period.

### 2.6. Statistical Analysis

Differences in distribution of background characteristics, nutrient and food intake by TRF status were assessed to identify potential confounding factors. Categorical variables are expressed as frequencies of occurrence and percentages, while continuous variables are expressed as means and standard deviations (SDs). The chi-squared test was used to assess the differences between groups. The Student’s *t*-test was used to assess the differences between groups with normally distributed variables, while the Mann–Whitney U test was used for not-normally distributed variables. The association between TRF status and metabolic outcomes was tested through univariate (unadjusted) and multivariate logistic regression analysis adjusted for baseline characteristics (including age, sex, educational and occupational status, smoking and physical activity status). An additional model was performed further adjusting for the level of adherence to the Mediterranean diet (as a proxy for diet quality) and having breakfast and dinner. All reported *p* values were based on two-sided tests and compared to a significance level of 5%. SPSS 17 (SPSS Inc., Chicago, IL, USA) software was used for all the statistical calculations.

## 3. Results

The main background characteristics of the patients by categories of time feeding are presented in Table 1. The 14.3% (*n* = 276) and 5.6% (*n* = 108) of individuals consumed their meals within 10 h (TRF-10) and 8 h (TRF-8), respectively. There was no significant different frequency of TRF between men and women; in contrast, among those categorized as TRF-10 and TRF-8, there were significantly older individuals and a higher percentage of subjects with medium educational and occupational status. Additionally, for physical activity, there was a significant difference in the distribution of categories for TRF-8, with a higher proportion of moderately active individuals among those eating within 8 h. When considering health status, there was a significant difference in BMI for individuals consuming their meals within 10 h and 8 h compared to those who did not. Additionally, a significant difference in systolic blood pressure was found, but only among those with feeding restricted to 8 h. In general, no clear trends across categories of the investigated variables have been detected.

Table 2 shows micro- and macronutrients and major food groups consumption across time feeding groups in order to test whether there was any quantitative or qualitative dietary difference between groups. There was no different energy intake between either TRF-10 or TRF-8 groups and no TRF groups. In contrast, participants eating in a restricted window of time had significantly lower intake of vitamin C, E and consumed more nuts and dairy products than those with longer feeding time. Moreover, individuals belonging to the TRF-8 group also had a lower intake of fiber, potassium, fruit, legumes, and higher amounts of meat (total).

Table 3 shows the association between TRF and the various metabolic outcomes investigated. After adjustment even for potential confounding factors related to eating habits (including adherence to the Mediterranean diet, having breakfast/dinner), TRF-10 was inversely associated with being overweight/obese (OR = 0.05, 95% CI: 0.01, 0.07), hypertension (OR = 0.24, 95% CI: 0.13, 0.45), and dyslipidemias (OR = 0.26, 95% CI: 0.10, 0.63), while TRF-8 was associated only with being overweight/obese (OR = 0.08, 95% CI: 0.04, 0.15) and hypertension (OR = 0.33, 95% CI: 0.17, 0.60). No associations were found with type-2 diabetes.

## 4. Discussion

In the current study, we investigated the relationship between the time feeding period and various metabolic outcomes in a cohort of Mediterranean adults. There were no clear patterns of association between the feeding window and background variables (besides age) as well as no healthier nutrient/food intake in those individuals with shorter feeding time. However, based on multivariate logistic regression analyses, we observed that individuals consuming their food in a window of time limited to 8 or 10 h were less likely to be obese and overweight, to have hypertension, and those with TRF-10 were also less likely to have dyslipidemias. Interestingly, individuals with diverse feeding time habits did not differ in total calorie intake, nor other important nutrients that could confound the analyses, suggesting that the plausible effects of time-restricted feeding are not due to energy or nutrient differences between groups.

The effects of a reduction in the time feeding window on body weight and other metabolic outcomes in humans have been studied in only a few studies due to both the novelty of the topic and the difficulty of following a daily life TRF protocol in the case of randomized controlled trials. However, published scientific literature on increasing the temporal fasting window shows results in line with our findings concerning its potential role on body weight and produces comparable, if not superior, weight loss, when compared to continuous caloric restriction [22,23]. Among randomized controlled trials, Gill et al. [24] investigated the effects of 10-h TRF in overweight adults and reported a 4% weight decrease that was maintained for one year. Findings from other studies [25,26] showed that a 4 to 8 h time feeding window induces a spontaneous calorie restriction and significantly lowered fat mass without changing muscle mass in young resistance-trained men. Another recent study [27] investigated the effects of 12 weeks 8-h TRF protocol (ad libitum food intake, time-limited from 10:00 to 18:00 h) in comparison to a no-intervention historical control group on weight and metabolic disease risk factors in obese patients. They found that TRF patients had a significant decrease in systolic blood pressure and reduction in body weight by ~3% relative to a no-intervention historical control group. In this study, body composition, visceral fat mass and metabolic biomarkers (including diastolic blood pressure, LDL and HDL cholesterol, triglycerides, fasting glucose and insulin, HOMA-IR (Homeostasis-Model Assessment-Insulin Resistance), and homocysteine) were not significantly different from controls. Lowe et al., comparing a consistent meal timing group instructed to eat three structured meals per day and a TRF group instructed to eat ad libitum from 12:00 p.m. until 8:00 p.m., showed that there was a significant decrease in weight in the intervention but not in the control group, nor between groups [28]. Conversely, Sutton [29] and colleagues performed a five-week crossover, randomized, isocaloric- and eucaloric-controlled feeding trial testing 6-h TRF in prediabetic male patients. Interestingly, TRF ameliorates insulin sensitivity, b-cell responsiveness, oxidative stress and blood pressure in prediabetic patients, even though food intake was matched to the control group and no weight loss occurred. Finally, a 12-week non-randomized controlled clinical trial performed in obese women showed that a TRF protocol reduces body weight without changes in metabolic syndrome biomarkers [30]. Overall, the major limitation of these studies relies on general inclusion of a few patients (maximum 40), only short-term interventions (maximum nine weeks) and incomparable meal frequency range (from 1 to 12 meals/day).

Concerning observational studies, a report conducted on 50,660 adult Canadian and US members of Seventh-day Adventist churches showed that changes in BMI were associated with the duration of the overnight fasting window: the longer the overnight fast, the lower the bodyweight [31]. A cross-sectional analysis conducted on overweight/obese women who were dependents of active duty and retired military personnel showed that TRF was associated with lower dietary glycemic load, energy intake and meal frequency [32]. Orthodox religious fasting and Ramadan can be also considered forms of TRF since food intake is only allowed at certain times of the day. Some studies showed that TRF associated with Orthodox fasting and Ramadan led to better body composition and a reduced risk of become obese [33,34,35], although other studies reported inconsistent results, often transient and heterogeneous [36,37]. However, there are some issues that do not allow to appropriately compare religious fasting to TRF regimens: the light/dark cycle of fasting and eating is often shifted as compared to natural circadian rhythms, the length of fasting window varies depending on geographical location and year, and various implementations of Ramadan fasting exist.

The exact mechanisms by which intermittent fasting and especially TRF can affect human health are still poorly understood, even though considerable progress has been made in our understanding. The fasting period associated with TRF can restore and amplify the peripheral circadian clocks and the transcription rates of clock genes [38,39] that regulate many metabolic pathways, which are altered in metabolic disease and obesity [40,41]. Eating in a shorter feeding time window seems to ameliorate the circadian oscillations of the main metabolic regulators, including cAMP response element-binding protein, mammalian target of rapamycin, and AMP-activated protein kinase [42]. Moreover, TRF may play a role in the pathophysiology of obesity through the modulation of the expression of protein chaperones (e.g., heat-shock proteins) and growth factors (e.g., brain-derived neurotrophic factor) that lead both to less oxidative damage and higher stress resistance [43], and, consequently, improved adipose tissue signaling and a lower increase of fat storage [44]. Additionally, a relationship between TRF and gut microbiota has been related to the potential effects on metabolic health through inflammatory and immune-related mechanisms [45]. Finally, although the duration of fasting during a TRF approach does not result in ketosis, if the fasting time window is prolonged (to about 16 h), it is sufficient to activate autophagy [46]. Autophagy is an important intracellular process that has a key role in the cardiovascular system to fight against oxidative stress and to maintain normal cell function [47].

Other metabolic outcomes including hypertension and dyslipidemias were associated with TRF when the analyses were not adjusted for diet quality (evaluated as adherence to the Mediterranean diet) and having breakfast or dinner. Thus, the association between TRF and overall metabolic health outcomes might be confounded by such variables. According to recent scientific literature, these variables may possibly be interconnected: several studies have been published on chronotype and cardio-metabolic outcomes, showing a substantial association of adverse outcomes with evening chronotype [48,49], including having dinner and higher energy intake during the evening [50,51]. Similarly, later chronotype has been also associated with breakfast skipping and both contribute to poorer glycemic control [52]. Concerning diet quality, several studies showed a relationship between higher adherence to the Mediterranean diet, morning chronotype, and health outcomes [53,54]. Although reducing the feeding time window during the day may be beneficial, shifting food intake to the darkest hours can partially reverse circadian rhythms and have null, if not detrimental, effects on metabolic outcomes; there is a large body of scientific literature showing that individuals consuming a larger daily caloric intake during the evening are more likely to be overweight and obese [55,56,57,58,59,60]; moreover, other studies showed that nocturnal eaters had an increased coronary heart disease risk [61] and reduced insulin sensitivity assessed by HOMA-IR index [62]. Notably, there are also contrasting results reported in the literature and other studies revealed that the time of eating was not predictive of responsiveness to weight loss interventions [63]. From a mechanistic point of view, eating in misalignment with circadian clocks may have an impact on hormonal and metabolic homeostasis, particularly glucose tolerance, cortisol levels, and adiponectin fluctuation [64,65,66]. Thus, the time of TRF (i.e., early or late TRF) may result in different outcomes when exploring its association with metabolic health, but as the results are not univocal, they warrant clarification in future studies.

The findings of the current study have to be considered in light of some limitations. First, the cross-sectional design of the study does not permit the definition of a causal relationship but rather an association. Second, data from an FFQ may be subject to recall bias. Third, we can only rely on average eating habits, but daily fluctuations of feeding times should be taken into account. Fourth, we were unable to distinguish early and late TRF due to the low number of individuals included in this category. Fifth, the low number of both individuals having TRF and type-2 diabetes may limit the statistical power for the retrieved lack of association.

## 5. Conclusions

In conclusion, metabolic disorders could not only be a matter of excess calorie intake but also considered a “chronobiological disease”, suggesting that differences in feeding/fasting duration could play a role in daily habits of the general population. Considering the results of the present study, time-restricted feeding could be implicated in the prevalence of metabolic outcomes such as being overweight/having obesity and having hypertension. However, these findings require further investigation, with studies that are better designed, more controlled, and with a prospective approach. Moreover, given the potential importance of distinguishing between early and late time-restricted feeding, it is advised that future studies take into account this variable when exploring the relationship between TRF and metabolic health outcomes.

## Figures and Tables

**Table 1:** Background characteristics of the study population according to time-restricted feeding duration.

	TRF-8				TRF-10			
	Yes (*n* = 108)	No (*n* = 1828)	*p*-Value *	*p_for trend_* *	Yes (*n* = 276)	No (*n* = 1660)	*p*-Value *	*p_for trend_* *
Sex, *n* (%)			0.369	-			0.121	-
Men	47 (43.5)	757 (41.4)			124 (44.9)	680 (41.0)		
Women	61 (56.5)	1071 (58.6)			152 (55.1)	980 (59.0)		
Age groups, mean (SD)	54.7 (15.7)	48.1 (17.6)	<0.001	-	49.2 (17.5)	43.8 (17.1)	<0.001	-
Educational status, *n* (%)			0.003	0.342			<0.001	0.381
Primary/secondary school	43 (39.8)	654 (35.8)			72 (26.1)	625 (37.7)		
High school	51 (47.2)	669 (36.6)			127 (46.0)	593 (35.7)		
University	14 (13.0)	505 (27.6)			77 (27.9)	442 (26.6)		
Occupational status, *n* (%)			<0.001	0.421			0.062	0.623
Unemployed	10 (11.0)	451 (28.8)			49 (21.3)	412 (28.9)		
Unskilled workers	30 (33.0)	236 (15.1)			46 (20.0)	220 (15.4)		
Partially skilled workers	30 (33.0)	410 (26.2)			67 (29.1)	373 (26.1)		
Skilled workers	21 (23.1)	470 (30.0)			68 (29.6)	423 (29.6)		
Smoking status, *n* (%)			<0.001	-			<0.001	-
Never smoker	43 (39.8)	1152 (63.0)			127 (46.0)	1068 (64.3)		
Former smoker	52 (48.1)	413 (22.6)			126 (45.7)	339 (20.4)		
Current smoker	13 (12.0)	263 (14.4)			23 (8.3)	253 (15.2)		
Physical activity level, *n* (%)			0.047	0.527			0.149	0.521
Low	27 (26.0)	302 (18.6)			42 (15.6)	287 (19.7)		
Moderate	40 (38.5)	816 (50.2)			132 (48.9)	724 (49.7)		
High	37 (35.6)	506 (31.2)			96 (35.6)	447 (30.7)		
Health status, *n* (%)								
Hypertension	49 (45.4)	927 (50.7)	0.281	-	95 (34.4)	881 (53.1)	<0.001	-
Diabetes	9 (8.3)	137 (9)	0.429	-	9 (8.3)	137 (9.0)	0.004	-
Dyslipidemias	11 (10.2)	345 (18.9)	0.012	-	15 (5.4)	341 (20.5)	<0.001	-
Overweight/obesity	21 (24.1)	926 (54.1)	<0.001	-	61 (27.0)	886 (56.4)	<0.001	-
BMI, mean (SD)	23.2 (2.4)	26.0 (4.62)	<0.001	-	23.5 (2.7)	25.9 (4.6)	<0.001	-
Systolic blood pressure	117.2 (11.6)	122.1 (12.9)	<0.001	-	122.3 (13.9)	121.7 (12.8)	0.606	-
Diastolic blood pressure	73.4 (8.8)	75.3 (10.6)	0.042	-	76.6 (11.1)	75.0 (10.4)	0.135	-

* Differences between groups were tested with Chi-square test.

**Table 2:** Mean (and standard deviation) of micro- and macronutrients and major food groups intake according to time-restricted feeding duration.

	TRF-8			TRF-10		
	Yes (*n* = 108)	No (*n* = 1828)	*p*-Value *	Yes (*n* = 276)	No (*n* = 1660)	*p*-Value *
	Mean (SD)			Mean (SD)		
Energy intake (kcal/day)	2106.3 (813.6)	2243.5 (885)	0.090	2104 (819.8)	2173.8 (806.8)	0.189
Energy intake (kJ/day)	8539.5 (3369.9)	9135 (3678.6)	0.076	8528.9 (3393.3)	8836 (3361.5)	0.163
Macronutrients						
Carbohydrates (g/day)	306.6 (127.4)	334.7 (132.7)	0.027	306 (127.9)	321.2 (127.0)	0.067
Fiber (g/day)	33 (17.7)	40 (26.1)	<0.001	33.2 (18.1)	35.2 (19.4)	0.098
Protein (g/day)	88.2 (37.8)	93.7 (47.6)	0.151	88.3 (38.8)	89.3 (36.3)	0.699
Fat (g/day)	62.5 (27.7)	63.6 (31.4)	0.682	62.3 (27.6)	64.1 (30.0)	0.315
Cholesterol (mg/day)	200 (109.7)	196.9 (140.6)	0.772	201.4 (112.6)	191 (105.3)	0.152
SFA	24.6 (11.4)	24.5 (12.2)	0.954	24.5 (11.2)	25 (12.8)	0.433
MUFA	26.3 (10.9)	27 (12.9)	0.475	26.2 (10.8)	27.1 (12)	0.212
PUFA	11.48 (5.9)	12 (6.0)	0.412	11.5 (6.0)	11.8 (5.3)	0.420
Total Omega-3	1.73 (0.9)	1.73 (1.0)	0.924	1.74 (0.9)	1.66 (0.7)	0.207
Micronutrients						
Vitamin A (Retinol)	5.8 (6.5)	6.6 (9.2)	0.119	897.4 (482)	904.2 (421.2)	0.825
Vitamin C (mg/day)	164.2 (115.1)	208.9 (154.8)	<0.001	164 (117.4)	182.8 (121.3)	0.014
Vitamin E (mg/day)	9 (4.0)	10 (4.8)	0.009	9 (4.0)	9.6 (4.2)	0.017
Vitamin B12	7 (7.4)	6.7 (6.5)	0.692	7 (7.7)	6.2 (4.8)	0.076
Vitamin D	894.3 (472.6)	967.5 (490)	0.220	5.9 (6.7)	5.5 (6.3)	0.267
Sodium (mg/day)	2937 (1208.1)	2824.2 (1246.4)	0.347	2907.6 (1191.6)	3069.5 (1310.6)	0.040
Potassium (mg/day)	3828.7 (1822.8)	4350.6 (2556.5)	0.005	3842.9 (1876)	3947.7 (1864.5)	0.390
Foods						
Cereals (total, g/day)	219.9 (138.1)	240.1 (127.8)	0.138	219.1 (138.7)	232.5 (130.2)	0.135
Vegetables (g/day)	272.2 (184.6)	296 (229.7)	0.201	273.5 (190.0)	273.5 (171.8)	0.999
Fruit (g/day)	407.4 (347)	542.4 (438.9)	<0.001	410.7 (352.6)	440.3 (362)	0.199
Legumes (g/day)	38.3 (49.8)	53.4 (73.0)	0.003	38.3 (51.3)	43.8 (52.4)	0.099
Nuts (total, g/day)	23 (43.0)	15 (19.0)	0.068	23.7 (47.9)	16.2 (19.3)	0.011
Fish (g/day)	69.1 (83.2)	77.3 (99.5)	0.326	70.5 (86.3)	64.2 (69.8)	0.247
Meat (total, g/day)	71.9 (40.3)	62.8 (34.9)	0.022	71.6 (39.7)	70.1 (42.2)	0.578
Red meat (g/day)	34.8 (26.7)	30.2 (22.0)	0.076	34.7 (26.9)	33.9 (23.4)	0.642
Processed Meat (g/day)	17.3 (21.0)	15.6 (28.0)	0.4	17.1 (21.3)	17.8 (22.5)	0.655
Dairy products (g/day)	201.6 (180.5)	165.9 (191)	0.047	206 (181.2)	160.8 (176.9)	<0.001
Alcohol (total, g/day)	7.6 (11.9)	7.5 (12.0)	0.953	7.7 (12.0)	6.8 (11.8)	0.256
Coffee (mL/day)	59.1 (45.6)	58.4 (47.5)	0.878	59.3 (45.7)	57.8 (45.9)	0.638
Tea (mL/day)	72.5 (146.5)	93.5 (164.0)	0.160	72.8 (149.3)	78.7 (137.0)	0.548
Olive oil (mL/day)	7 (3.2)	7 (3.2)	0.802	7 (3.2)	7 (3.2)	0.746

* Differences between groups were tested with Student’s t-test or Wilcoxon–Mann–Whitney U test.

**Table 3:** Association between time-restricted feeding duration and main outcomes of interest in the study population.

	OR (95% CI) *			
	Overweight/Obesity	Hypertension	Type-2 Diabetes	Dyslipidemias
TRF-8				
Model 1	0.27 (0.16, 0.45)	0.80 (0.55, 1.19)	1.12 (0.56, 2.27)	0.48 (0.26, 0.92)
Model 2	0.19 (0.11, 0.32)	0.46 (0.29, 0.72)	0.72 (0.34, 1.53)	0.34 (0.17, 0.65)
Model 3	0.20 (0.11, 0.37)	0.37 (0.22, 0.63)	0.78 (0.35, 1.75)	0.25 (0.12, 0.53)
Model 4	0.08 (0.04, 0.15)	0.33 (0.17, 0.60)	1.03 (0.38, 2.87)	0.46 (0.19, 1.15)
TRF-10				
Model 1	0.30 (0.21, 0.41)	0.46 (0.35, 0.60)	0.37 (0.18, 0.74)	0.22 (0.13, 0.37)
Model 2	0.44 (0.30, 0.66)	0.52 (0.38, 0.71)	0.46 (0.22, 0.95)	0.26 (0.14, 0.45)
Model 3	0.33 (0.23, 0.48)	0.42 (0.30, 0.60)	0.48 (0.22, 1.02)	0.20 (0.11, 0.38)
Model 4	0.05 (0.01, 0.07)	0.24 (0.13, 0.45)	0.59 (0.21, 1.64)	0.26 (0.10, 0.63)

* Associations were tested with logistic regression analyses. Model 1 unadjusted. Model 2 includes age and sex. Model 3 includes variables as model 2 + educational and occupational level, smoking status, and physical activity level. Model 4 includes variables as model 3 + energy intake, Mediterranean diet adherence score, breakfast (yes/no) and dinner (yes/no). NA, not applicable.
